# The Real-Time Comprehension of Idioms by Typical Children, Children with Specific Language Impairment and Children with Autism

**DOI:** 10.4172/2472-5005.1000130

**Published:** 2017-12-15

**Authors:** Matthew Walenski, Tracy Love

**Affiliations:** 1 Department of Communication Sciences and Disorders, Northwestern University, USA; 2 School of Speech Language and Hearing Sciences, San Diego State University, USA; 3 Center for Research in Language, University of California San Diego, USA

**Keywords:** Autism, Children, Language, Language disorders, Specific language impairment

## Abstract

**Objective:**

We examined on-line auditory idiom comprehension in typically developing (TD) children, children with specific language impairment (SLI), and children with autism. Theories of idiom processing in adults agree on a reliance on lexical/semantic memory for these forms, but differ in their specifics. The Lexical Representation hypothesis claims that literal and non-literal meanings are activated in parallel. The Configuration hypothesis claims that a non-literal meaning will take precedence, such that a literal meaning may not be activated at all.

**Method:**

Children aged 6–16 years listened to sentences containing idioms for a cross-modal priming task. The idioms were ambiguous between an idiomatic and a literal meaning. We looked at priming for both meanings at the offset of the idiom.

**Results:**

TD children (n=14) and children with SLI (n=7) primed for the idiomatic but not literal meaning of the idiom. Children with autism (n=5) instead primed for the literal but not idiomatic meaning.

**Conclusions:**

TD children showed an adult-like pattern, consistent with predictions of the Configuration Hypothesis. Children with SLI showed the typical pattern, whereas the atypical pattern observed for children with autism may reflect a particular deficit with complex material in semantic memory.

## Introduction

Figurative language, including sarcasm, irony, and the use of idiomatic expressions, is pervasive in everyday speech, such that successful language comprehension requires facility with these forms of non-literal expression. In the current paper we focus on the processing of sentences containing idioms; multi-word phrases with meanings that generally can’t be predicted by the meanings of the individual words in the phrase [1]. For example, *spill the beans* has an idiosyncratic interpretation of “reveal a secret” that is not predictable from the meanings of the individual words. Idiom meanings therefore must be specified in the mental lexicon, like the meanings of typical words. However, idioms are unlike typical words in that they are syntactically structured – *spill the beans* consists of a verb *spill* and its direct object the *beans*.

According to the Lexical Representation Hypothesis [2], the meaning of an idiom is stored in the lexicon like that of other words. During comprehension, an idiomatic meaning will be accessed in parallel with the literal meanings of the individual words in the phrase [3,4]. Thus for an idiom like *spill the beans*, the meaning of /beans/and the idiomatic meaning /reveal secrets/should both be active as the word beans is processed. According to the Configuration Hypothesis [5] an idiom’s meaning is associated with a configuration of its constituent words, and becomes available during comprehension as sufficient input accumulates to recognize the configuration. A key difference between these hypotheses is that according to the Configuration Hypothesis, predictably configured idioms won’t activate the literal meanings of the constituent words, and may actually inhibit them [5,6]. On this view, only a meaning related to *revealing secrets* should be accessed during comprehension of *spill the beans*, not a meaning related to beans as a food item.

Importantly, both hypotheses posit that the meanings of idioms are represented in the mental lexicon, which is itself posited to depend on declarative/semantic memory [7]. Thus on either hypothesis successful comprehension of the meaning of an idiom should engage lexical knowledge in semantic memory [8,9]. Idiom comprehension is therefore expected to be atypical in children with disordered memory systems. In this study we investigate the activation of idiomatic and literal meanings of idioms during real time sentence comprehension in typically-developing children and in children with developmental disorders that impact language and memory.

For typical language development, evidence suggests a late developmental course, with children not achieving adult-like performance with figurative language until age 11 or so [10–12]. However, investigations into the development of idiom comprehension have relied principally on two types of tasks: definition tasks, where an idiom is provided and the child is asked to define or explain it, and multiple choice tasks, where the child is given an idiom and asked to match it to the best choice from an array of written meanings or pictures [13]. Both tasks constitute off-line measures.

Off-line methods such as these are temporally insensitive and allow, encourage, or even require conscious, metalinguistic reflection on the aspects of language being tested. Thus results from off-line tasks reflect quite different processes than are called on during automatic language comprehension [14]. In contrast, temporally sensitive “on-line” methods are capable of discriminating early processes that are precursors to the final output of processing (e.g., conscious apperception of the meaning of an idiomatic phrase) during auditory language comprehension. These early processes are rapid, automatic, and shielded from conscious inspection [15,16].

Crucially, evidence suggests that automatic language processing is adult-like in children at a young age, developing earlier than an off-line ability to think about language. Using an on-line cross-modal priming task, Love, Walenski, and Swinney [16] found a clear dissociation in young children’s abilities to process (real time) and interpret (off-line) sentences containing reference-seeking elements such as pronouns as seen in sentence.

(1) The *bird* says that the turtle with the hard shell is rubbing *him* with suntan oil on the sandy beach.

In this study, children as young as 5 years old successfully linked the pronoun (*him* ) with its antecedent (*the bird* ) immediately after hearing him during the uninterrupted auditory sentence. In an off-line sentence-picture matching task however, those same children who were under 8 years of age were unable to reliably identify the antecedent (for a similar finding, see Ref. [17]). Similarly, adult-like formation of dependency links in real time has been demonstrated with other complex sentence constructions as well. In one study, a group of 4–6 year old children showed adult-like on-line comprehension of relative clause structures; [18]; in another, a group of 5–12 year olds showed adult-like on-line comprehension of verb-phrase ellipsis constructions [19].

With respect to idioms, we therefore hypothesize that typically-developing children have an adult-like on-line awareness of an idiom’s meaning from a young age, even if the metalinguistic skill to correctly indicate comprehension in an off-line task is slower to develop. However, children with developmentally disordered language may show aberrant on-line processing of idioms, whether or not off-line performance appears typical. In the current study we examine two such disorders: Specific Language Impairment and Autism.

*Specific Language Impairment (SLI)* is a developmental disorder defined by language impairments in the absence of any clear mental or physical handicap or frank neurological damage [20,21]. Children with SLI form a heterogenous group, though structural language impairments are generally more severe than lexical level dysfunction [21,22]. Moreover, children with *SLI* may compensate for at least some structural deficits by storing larger units as lexical chunks in declarative (semantic) memory [21]. Thus as successful idiom comprehension depends on an intact lexical/semantic memory [8,9], comprehension of an idiom’s meaning may be age-appropriate in children with SLI [11,12].

Prior studies of idioms in SLI reveal strong performance with forced choice comprehension tasks, in which the experimenter provides pictures or definitions and the child chooses the best match for an idiom [23,24], though not in all studies [25]. However, children with SLI frequently perform poorly on idiom definition tasks, whose difficulty is exacerbated by a reliance on structural language to craft the response [23,26,27], though again, not in all studies [27,28]. Impairments are also seen with non-verbal play acting tasks, in which children use toys to act out the meaning of an idiom [13]. Thus whether performance is spared or impaired appears to be at least partly task-dependent. However, no studies we are aware of have examined idiom comprehension in SLI with an on-line task.

*Autism* is a developmental disorder that is characterized by abnormal social interaction, abnormal language and communication, and restricted and repetitive behaviors and interests [29]. Impairments of structural language are also found, though not in all individuals [30–35]. In contrast, despite a great deal of heterogeneity and delays in language development in many individuals, vocabulary is often a relative strength in the disorder, even if not necessarily “normal” in all respects [30,36–40]. As well, performance at tasks involving previously-learned single words, in both receptive and expressive domains, can to be relatively spared in many individuals, and may even be enhanced in some respects, particularly for high-functioning individuals [31,41–43].

Difficulty with figurative language is a hallmark feature of autism [44], with common anecdotal reports of overly literal interpretations of idioms. Consistent with these anecdotal accounts, idiom deficits are observed in children and adults with autism with off-line measures, with definition tasks [13,26,28,45,46], forced choice recognition tasks [45,47–50], play-acting [13,28], and a meaningfulness judgment task [51], though typical performance was seen in a story-based truth judgment task [52]. Consistent with a comprehension deficit, responses indicative of literal interpretations are often found [48,50]. Several hypotheses have been put forward to explain deficient idiom comprehension in autism, including that it reflects a deficit of pragmatic language and an inability to recognize speaker intent [53]. Alternatively, it has been suggested that there is no deficit specific to idioms, but that any such apparent deficits reflect non-specific difficulties related to overall language competence [26,54].

As mentioned earlier, most prior studies of idiom comprehension have relied on off-line tasks, which are not temporally sensitive. Indeed, on-line studies reveal that pragmatic inferences take several hundred milliseconds to develop [14]. Therefore, if deficient idiom comprehension reflects deficient inferential or contextual processing in autism, a deficit may be found with off-line measures, even if idiomatic meanings are intact and initially accessed normally. However, results from an on-line event-related potential study of idiom comprehension, in adults with autism, indicate abnormal N400 amplitude in an idiom recognition task [51]. Tellingly, the N400 component is an index of the state of semantic memory [55], suggesting that deficits in idiom comprehension in autism could reflect a semantic memory deficit [56], despite apparently strong semantic memory abilities in high-functioning individuals, at least with simple tasks [57–60].

In the current study we use cross-modal priming to examine children’s on-line comprehension of familiar idiom phrases embedded in sentence contexts. We test for priming of idiomatic and literal meanings at the offset of an idiom in an auditory sentence in three groups of children: Typically developing children, children with specific language impairment, and children with autism. Note that group comparisons are not made directly – all of our hypotheses are examined with within-group analyses only.

For *typically developing children*, we hypothesize that on-line idiom processing is adult-like from an early age, mirroring previous findings from pronouns and verb-phrase ellipsis constructions, where on-line processing was adult-like, and had developed at an earlier age than offline comprehension performance. If on-line idiom processing is adult-like, the lexical representation hypothesis predicts priming for both the literal and idiomatic meanings. In contrast, the configuration hypothesis predicts priming for the idiomatic but not literal meaning. Otherwise, if on-line idiom comprehension is not yet adult-like, we may expect to see activation only for the literal meaning.

For *children with SLI*, if on-line idiom comprehension depends on structural language ability, then deficient processing of the idiomatic meaning is expected. If on-line idiom comprehension depends only on lexical (semantic) memory, then on-line performance might be similar to that of the typical children, though it might also be impaired if the children present with a lexical deficit relative to their typical peers.

For *children with autism*, if deficient (final) idiom comprehension reflects deficient pragmatic or context-based processing, then we expect immediate (on-line) processing of an idiom might still be consistent with the typical pattern. Alternatively, if there is a semantic deficit in autism, then immediate on-line priming for the idiomatic meaning is not expected.

## Method

### Participants

We tested thirty-nine right-handed native monolingual English speaking children aged 6–16 years with normal or corrected to normal vision and hearing, and average or above-average non-verbal intelligence, based on the Test of Non-Verbal Intelligence-3 (TONI-3) [61]. All children were reported to be able to read, minimally, at their grade level. Children were divided into three groups for this within-subjects investigation based on whether they were typically developing (n=25) or had a diagnosis of specific language impairment (SLI, n=8) or a diagnosis of autism (n=6). Thirteen participants were excluded due to poor performance on the button press decisions (lower than 75% accuracy for typically-developing children, n=11; lower than 60% for the two other groups: 1 child with SLI, 1 child with autism). Demographic data for the remaining 26 children are given in Table 1.

Diagnosis of language impairment was based on the Clinical Evaluation of Language Fundamentals-IV (CELF-IV)[62] and defined as a score at least 1 standard deviation below the mean on two of the four core language subtests [63,64]. In addition, each child was tested on the Peabody Picture Vocabulary Test (PPVT-3) [65], and the Test of the Reception of Grammar (TROG-2)[66], to assess the presence of language impairment in these domains.

Diagnosis of autism was made by a licensed clinician on the basis of DSM-IV criteria, per the Autism Diagnostic Interview-Revised (ADI-R) [67] and The Autism Diagnostic Observation Schedule (ADOS-G) [68]. Children with known etiology of autistic symptoms (e.g., Fragile X syndrome) were not included. As an additional measure, the Social Interaction Difference Index (SIDI) score from the Children’s Communication Checklist (CCC-2) [69,70] was computed for each child in the SLI and autism groups (Table 1) (note that a score was not available for one child with SLI). The SIDI score is a calculated measure from scaled scores on the CCC-2. Positive scores indicate greater non-pragmatic language impairment; negative scores a greater pragmatic and social impairments.

Children with no language impairment constituted the typically-developing group. Children in the SLI and typically-developing groups were free of autism and did not have immediate family members diagnosed with autism. The same language criteria were applied to the autism and SLI groups, to identify children in either group with language impairment(s) in any of these areas. One child with autism also met criteria for language impairment. Two children with SLI had co-morbid Attention Deficit Hyperactivity Disorder (ADHD); the remaining children were free of any other developmental, neurological, or psychiatric disorders.

To complete the experiment, each child returned for four visits, each lasting approximately 1 hour. After each visit, parents of participants were compensated $5 for traveling expenses, and the children were allowed to choose a prize. Upon completion of the study, each participant was given an additional $25. Participants were recruited from schools in the San Diego Unified and Del Mar School Districts. Parents and children were informed about the study and gave informed consent. This experiment and all the following experiments were conducted with the approval of the Institutional Review Boards at the University of California San Diego and San Diego State University.

## Materials and Design

We used a Cross-Modal Lexical Priming task (CMLP)[15,71], in which children listened to sentences over headphones while monitoring a computer screen for a letter-string visual target that appeared at a specific moment in the sentence. Children decide by button press whether the visual target is a word (right button) or non-word (left button). The underlying assumption behind this task is that participants will respond more rapidly to visual stimuli that are related conceptually to elements in the auditory sentence due to unconscious priming effects, thus providing an index for implicit comprehension of the literal and idiomatic meanings of idiomatic expressions.

To create the experimental sentences, we selected 40 three-word idioms that consist of a verb and a noun phrase (e.g., “bury the hatchet”) and that are ambiguous between an idiomatic meaning and a plausible literal meaning. We did not include idioms for which the literal meaning would have been impossible (e.g., “walking on air”) or awkwardly phrased (e.g., “make a killing”), or that contained the pronoun ‘it’ (e.g., “ride it out”). Additionally, as our purpose is to investigate the separate availability of the literal and idiomatic meanings of these idiomatic phrases, we avoided idioms for which these meanings were too similar (e.g., “lend an ear”). The 40 idioms were each incorporated into a sentence biased towards the idiom’s idiomatic meaning, as an example (2). Each sentence consisted of a subject, the idiom (bolded in the example), a three word phrase that disambiguated the idiom towards its idiomatic meaning (underlined in the example), and a concluding phrase. The beginning of each sentence was made contextually neutral, to avoid biasing the sentence towards either meaning prior to the disambiguating phrase.

(2) The little girl with lots of freckles **dished the dirt** * about the secret in front of all her friends and everyone was very surprised.

Literal Related: **soil**Literal Control: **belt**Idiomatic Related: **gossip**Idiomatic Control: **duty**

**Figure 1:**
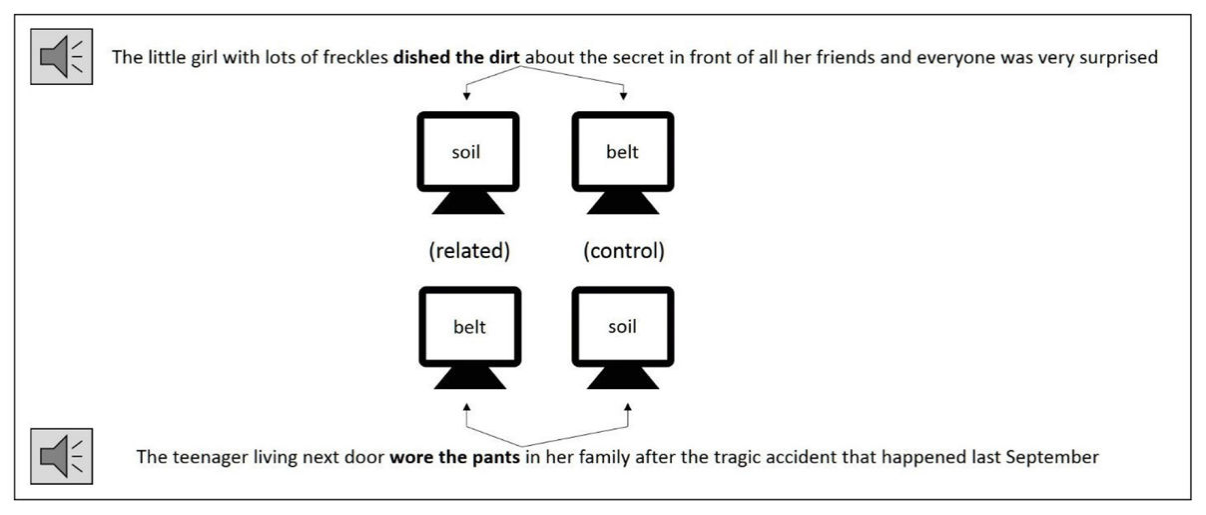
Example of two sentences with switched related and control targets for the literal meaning of the idiom phrase (in bold).

For each sentence, we chose four visual target words: One target was a semantic associate of the idiom’s literal meaning (but was not associated with the idiomatic meaning), one target was semantically related to the idiomatic meaning of the idiom (but was not related to the literal meaning), and two targets served as unrelated control targets. We used a matched sentence design, such that the related targets for one sentence served as the control targets for another, and vice versa (Figure 1). Over the full set of items, the related and control targets for each meaning were therefore perfectly matched to each other for length, frequency, etc., as the two sets consisted of exactly the same words. All of the targets for the experimental items were real English words, and were presented immediately at the offset of the idiom (i.e., at the position marked by the * in example 2).

The sentences were recorded by a female native English speaker at a normal rate of speech (mean: 4.75 syllables per second, SD: 0.39; range: 3.93–5.82) [72–74]. Forty filler sentences were created to vary the sentence structure used throughout the stimuli (10 contained idioms; 30 did not), to balance the number of word/non-word responses (40 of each), and to vary the timing of when the visual target appeared during the sentence.

Experimental and filler items were intermixed and pseudo-randomly ordered into a single script such that no more than 3 word or non-word targets appeared in a row. As we tested for priming of the two meanings, with related and control targets for each meaning, we had a 2 (meaning: literal, idiomatic) × 2 (target: related, control) matched sentence design (the control targets for one sentence were the related targets for another). Therefore, the experimental items were counterbalanced across four test scripts such that participants were exposed to items in every condition in each script, but participants did not hear a given idiom more than once per script. This was a fully within-subjects design, with every participant completing all four scripts, in separate sessions at least one week apart.

### Materials pretests

In order to ensure that we chose familiar idioms, and to ensure that the targets were appropriately related or unrelated, we conducted two pretests of an initial set of 60 idioms and their target words with two groups of college-age students.

#### Familiarity pretest

Twelve typical monolingual native English speaking UCSD undergraduates (age: 21.7 years; SD: 1.17; range: 20–24; all women; 10 right handed, 2 left handed) rated the familiarity of the 60 idioms on a five-point scale (1=not at all familiar; 5=highly familiar). Participants were instructed to rate how common or familiar they think the idiom is. The idioms were presented visually in their full idiom-biased sentences (as in example 2 above), and were underlined to ensure that participants knew which words to rate. Participants were also able to select “don’t know” instead of a rating, if they did not recognize the idiom or know its meaning. Results from this pretest are presented below.

#### Priming association pretest

In order to ensure the validity of the selected visual targets for use in the cross modal priming task, a separate group of 88 typical, right handed, monolingual native English speaking UCSD undergraduates (age: 19.8 years; SD: 1.73; range: 17–29; 27 male, 61 female) rated the strength of the association between the idiomatic and literal meanings of the 60 idioms and their related and control target words. Participants rated the associations between 1 (not at all related) to 5 (highly related), and were able to select ‘don’t know’ if they were unsure or did not know the meaning of the idiom. The idioms were presented in isolation (not in sentences), to ensure that participants rated target words in relation to the idiom alone, avoiding influence from the other words in the sentence.

We tested the relatedness of the related and control targets for each meaning of each idiom. In addition, to ensure that either the related or control targets for the idiomatic meaning weren’t associated with the literal meaning, and vice versa, we also tested the idiom targets with the literal meaning, and the literal targets with the idiomatic meaning. Thus we tested each meaning of each idiom with four target words. This was necessary to avoid a potentially serious confound – for example, if only the literal meaning were activated, but the target for the idiomatic meaning was also related to the literal meaning, then we might see priming for both meanings. Eight separate lists were compiled such that the four targets and two meanings for each idiom were counterbalanced across lists, with each idiom appearing only once per list. Each entry on the list specified whether the rating should be based on the literal or idiomatic meaning of the idiom (e.g., Literal meaning of: buried the hatchet). Each list was pseudo-randomized (no more than three of any type of item in a row) and contained all 60 idioms, 30 with literal and 30 with non-literal meanings, with 15 related and control targets for each meaning. Each participant saw only one list.

#### Pretest results

Of the 60 idioms included in the pretests, we excluded 16 for which the idiomatic meaning-related targets were at least weakly related to the literal meaning (mean rating above 2.8). One additional idiom was removed for having more than 20% “don’t know” responses on the familiarity pretest. Finally, three idioms were removed as we were unable to come up with a biasing context that clearly excluded the literal meaning.

The remaining 40 items were used in the experiment. These items had a mean familiarity rating of 4.45 (SD: 0.58, with all items above 3.0 except 1, which had a mean rating of 2.92), and an average of only 1% “don’t know” responses (SD: 3.2; only one item had 2 such responses). In addition, the related targets were related only to one meaning (targets related to the literal meaning were not related to the idiomatic meaning, and vice versa), and the control targets were unrelated to either meaning as well (Table 2).

## Procedure

Prior to beginning the experiment, the participants went through a four stage training and practice protocol to ensure both that they understood the dual nature of the task (listen to and comprehend the ongoing sentences; make a button-press decision to the pictures), and that they could perform the two tasks simultaneously. In *stage one* of the training, children were presented with visual targets (which were never the same as the targets used as experimental items) printed on index cards (11 words, 9 non-words) and asked to respond verbally with “Yes” if the letters were a real English word and “No” if the letters were not a real word. If the child made an error, the experimenter discussed the item with the child until they arrived at the correct identification. In *stage two*, the children were introduced to a two-button response box where the right button was labeled as “Yes” (i.e., word) and the left button was labeled as “No” (i.e., non-word). The button box was large enough that the children could rest their arms on it, with each hand dedicated to a specific button. The experimenter then presented the child with the same targets from the first stage and the child responded non-verbally, using the button box. If the child made an error, the experimenter again provided feedback. In *stage three*, the children viewed the targets on a computer screen (without sound) and responded using the button box. The children were instructed to respond as quickly as possible. The targets were presented in a series of blocks. In order to encourage speeded responses while maintaining high accuracy, each target remained on the screen for 1500 ms during the first block, but only remained on the screen for 500 ms in the following blocks. If this shorter presentation time caused any difficulties, feedback was provided during the pauses between blocks.

Finally, in *stage four*, the children were introduced to the cross-modal task. The children were told that they would be listening to sentences while watching the computer screen for the words. At specific moments during the uninterrupted sentence, a letter string target would appear on the screen and they had to use the button box to decide as quickly as possible whether the target was a “word” or “non-word”. During this stage, participants were trained to 100% button press response accuracy and also answered comprehension questions to ensure they understood the sentences. After the first visit, the training cards were no longer used, but the training script without sound was used to re-familiarize the children with the task.

Following successful completion of the four stages of training, participants began the actual experiment. Note that none of the items (visual targets, auditory sentences) that were used in any stage of training appeared as experimental stimuli; participants were never trained on the experimental items. An in-house software package (TEMPO v. 2.1.2) controlled stimulus presentation and data collection with millisecond accuracy. Each sentence was presented auditorily through headphones and the appropriate target was displayed on the computer screen. Response time measurement was initiated with the onset of the target and stopped by the button press response. Target presentation time was for a maximum of 1500 ms (but disappeared if a response was made before this maximum time was reached) and responses were collected for an additional 2000 ms, leaving a 3500 ms window within which a participant’s response would be recorded. Any response times longer than 3500 ms were recorded as a “no response” and counted as an error. There was a 3000 ms interval between successive sentences.

In addition to the experimental stimuli, sixteen multiple-choice comprehension questions were pseudo-randomly dispersed throughout the script. Questions were phrased such that only general topics were tested with the sole intention of reinforcing attention to the sentences. An experimenter read the questions at the proper points and recorded participants’ responses. Each test session lasted approximately 30 minutes.

## Analysis

Data from each participant group were analyzed separately. Children in all three groups (TD, SLI, autism) performed well on the task related comprehension questions (TD: mean=89.6%, SD=7.3; SLI: mean=80.2%, SD=13.6; Autism: mean=75.0%, SD=31.8), and responded accurately to the button-press decision task (TD: mean=90.5%, SD=5.6; SLI: mean=78.1%, SD=8.9; Autism: mean=91.0%, SD=7.8). Note that unlike for the button press decision (see Participants section above), no participants were excluded based on the comprehension questions, as these were merely to encourage attention to the auditory stimuli, and were not intended to accurately reflect comprehension. Prior to analysis data from two items (from one item in the literal condition, and from one item in the idiomatic condition) were removed from all groups, due to inadvertent priming between the targets and words in the sentence prior to the idiom, which might have therefore skewed the priming results.

For each analysis, correct responses were screened as follows: First, we removed response times less than 300 ms as being implausibly fast and thus likely button press errors. Second, extreme outliers (outside the outer fence of a box plot; any response farther than three times the interquartile range from the first and third quartiles) were identified for each group and removed. Third, we removed outliers more than 2.5 (TD) or 3.0 (SLI, autism) standard deviations from each child’s condition mean (meaning, relatedness). The looser screen for the children with SLI and children with autism was necessitated by the smaller data sets for those groups. In total, these screens removed a minimal amount of data: 2.4% of the TD data, 1.9% of the SLI data, and 1.5% of the autism data, distributed roughly equally across conditions for each group.

Analysis of the response times for each group was conducted using a mixed-effects restricted maximum likelihood (REML) regression model (SAS version 9.3, proc mixed) with crossed random effects on the intercept of Participant and Sentence, and fixed effects of meaning (literal *vs.* idiomatic), Relatedness (Control *vs.* Related targets), and their interaction. Response times were natural-log transformed for analysis. The models were fit with an unstructured covariance matrix for each random effect. Type III F-tests are reported for main effects and interactions. The fixed and random effect terms were entered into the model per our design and hypotheses, and so model fit was not used as criteria for inclusion of terms. To test the presence of priming for a particular meaning, we ran a priori planned comparisons of Related and Control targets for each meaning. For these comparisons, we computed t-tests of the differences of the least square means from the full model, and report the estimate of the difference (from in-transformed data), the standard error, t-value, 1-tailed p-value, and 95% confidence interval; note that p-values are otherwise reported 2-tailed. Degrees of freedom were computed using the Satterthwaite approximation.

## Results

Mean response times and standard error for each group and condition are given in Table 3. For the typically-developing children, *a priori* planned comparisons of the target response times revealed significantly faster responses for targets related to the idiomatic interpretation *vs.* their control probes (51 ms advantage; B=0.03 (0.02), 95% CI: [0.004, 0.06], t(1886)=2.19, p=0.01) but no advantage was seen for targets related to the literal interpretation *vs.* their control probes (-25 ms difference; B=0–0.01 (0.02), 95% CI: [−0.04, 0.02], t(1884)=0.72, p=0.24). The difference in the priming effects for the different meanings was reinforced by a significant interaction between Meaning and Relatedness (F (1,1880)=4.27, p=0.04). Main effects of Meaning and Relatedness were not significant (both ps>0.29).

To assess individual differences, we computed priming effect sizes for the figurative and literal meanings for each participant by recoding the related/control variable as 0 (related) or 1 (control) for each response, and correlating this new variable with response time using a standard Pearson correlation. The resulting r-value is an effect size, with values ranging from −1 (inhibition) to 1 (priming). While individual effect sizes were small (in the range of 0.1 for Pearson correlations)[75], 7 of the 14 children had an effect size greater than 0.1 for the figurative meaning, while only 1 of 14 had an effect size greater than 0.1 for the literal meaning. In addition, 11 of 14 children had a larger effect size for the figurative than literal meaning. Thus individual children largely conformed to the group pattern.

A similar pattern was seen for the children with specific language impairment, with significantly faster responses to related than control targets for the idiomatic interpretation (33 ms advantage; B=0.04 (0.02), 95% CI: [−0.004, 0.09], t(805)=1.81, p=0.04) but no advantage for targets related to the literal interpretation *vs.* their control probes (4 ms difference; B=−0.006 (0.02), 95% CI: [−0.05, 0.04], t(800)=0.26, p=0.79). Overall, there were no significant main effects or interactions (all ps>0.13). On an individual level, 4 of 7 children had a priming effect size greater than 0.1 for the figurative meaning, compared to 2 of 7 with an effect size of 0.1 or greater for the literal meaning. In addition, 4 of 7 children conformed to the typical pattern of a greater effect size for the figurative over the literal meaning.

However, a substantively different pattern was seen for the children with autism, who showed significant priming for the literal meaning (control-related: 44 ms; B=0.04 (0.02), 95% CI: [−0.006, 0.09], t (659)=1.72, p=0.04) but not for the idiomatic meaning (control-related: 10 ms; B=0.002 (0.02), 95% CI: [−0.05, 0.05], t (662)=0.07, p=0.47). There were no significant main effects or interactions for this group (all ps>0.21). Individually, 2 of 5 had a priming effect size greater than 0.1 for the literal meaning, while only 1 had an effect size greater than 0.1 for the figurative meaning. However, unlike for the other two groups, 4 of 5 children with autism had larger effect sizes for the literal than figurative meanings, with only one child showing the opposite, typical pattern.

## Discussion and Conclusions

In sum, the typically developing children activated the idiomatic but not literal meaning of the idiom phrase, immediately at the offset of the phrase. The children with SLI showed the same pattern as the typical children, with priming for the idiomatic meaning of the idiom phrase. In contrast, the children with autism primed only the literal meaning, not the idiomatic meaning. Importantly, the majority of individuals in each group showed the group pattern, suggesting that individual heterogeneity was not a determining factor in the results (though sample sizes for the SLI and autism groups were small, see below).

Turning first to the results from the typical children, the results are consistent with the predictions of the configuration hypothesis, and indicate that the children show an adult-like pattern. This also directly implies that the children have learned the meanings of the idioms (whether or not they would perform well on an explicit measure of idiom comprehension), as the meanings of the idioms were not predictable from the words in the sentences. The results do not appear consistent with the predictions of the lexical representation hypothesis, as priming should have been found on this view for the literal meaning of the phrase-final noun (e.g., *soil* should be primed at the offset of *dirt* in …*dished the dirt*…), contrary to our results.

These results apparently contradict the results of prior studies with off-line measures, which report a slower developmental course for idioms and adult-like performance only for older children (see Introduction). Given the broad age range of our participants, it is possible that the priming effects were due mainly to the older children. However, in follow-up analyses examining the effect of age on priming for each meaning separately (using the same regression approach described above, except that only relatedness, age, and their interaction were included as fixed effects), the priming effects did not change reliably as a function of age for either meaning (i.e., the interaction between Age and Relatedness was non-significant: Literal p=0.28; Idiomatic p=0.41), suggesting that this is not actually the case. Thus the results appear consistent with prior results of on-line processing developing in advance of off-line ability [16–19].

Children with specific language impairment showed the same pattern as the typically-developing children, with significant priming for the idiom’s non-literal meaning, but no priming at all for the literal meaning at the offset of the ambiguous idiom phrase. This suggests that the children with SLI had at least some representation of the meanings of the idioms, and that automatic processes of idiom comprehension are functioning age-appropriately in this population, despite their somewhat low single word comprehension scores (PPVT; Table 1). The results also suggest that prior reports of impairments at idiom comprehension (see Introduction) may have reflected particular difficulties with off-line language tasks, and not deficient processing of idiom meanings. Our results suggest that structural language ability is therefore not critical for successful on-line idiom comprehension.

For the children with autism however, a different pattern emerged, with children priming for the literal meaning of the phrase-final word, but not the non-literal meaning of the idiom. This is consistent with previous findings of deficient on-line and off-line performance with idioms, and a tendency to interpret idioms literally. Here we consider possible interpretations of this on-line performance.

First, the lack of priming for the idiomatic meaning might reflect a deficit of learning the arbitrary meanings of idiomatic expressions, perhaps reflecting a more general deficit related to word learning. That is, it might be that the children with autism never learned the non-literal meanings of these idioms. However, this is not likely given that that children with autism were native speakers of English with higher-than-normal verbal IQ scores, and higher-than-normal vocabulary scores (per the PPVT). Thus unless a word learning impairment is restricted to idioms, it seems unlikely that these meanings would not have been learned. However, children with autism were able to improve their performance on an idiom definition task after two weeks of targeted intervention in which they were explicitly taught the meanings of idioms [76]. In addition, it is also not likely that the deficit reflects a difficulty with processing multiple meanings of words. One study found that high-functioning children with autism (mean age 9 years) were not different than age-matched controls at providing two definitions for ambiguous words (e.g., *bat* ) and sentences (e.g., *don’t take my picture* ) [47].

Second, the lack of priming for idiomatic meanings is unlikely to reflect difficulty with pragmatic inferences. Pragmatic inferences take time to generate [14]. Thus if activation of an idiom’s non-literal meaning depends on such inferences, we would not expect to see immediate activation in the typical group, contrary to our results in that group. Therefore it is more likely that the lack of priming for an idiomatic meaning in autism reflects a temporally earlier process as well. Likewise, it is unlikely that the lack of priming reflects difficulty with structural language — only one of our participants met criteria for language impairments, and in any event the children with SLI, who all presented with structural language deficits, did show priming for the non-literal meaning.

A likely candidate is therefore a deficit of lexical/semantic memory, consistent with claims for low-functioning autism [56]. As well, studies of adults with autism using event-related potentials consistently report abnormalities of the N400 component [77,78], consistent with semantic memory dysfunction (on the interpretation of the N400 that it indexes semantic memory [55]). However, as performance with single words can be quite good in individuals with autism, and even better-than-normal in some respects [41,79,80], it may be that, at least in high-functioning autism, an idiom deficit reflects only certain aspects of semantic memory that are deficient in the disorder.

An additional issue however is that evidence suggests that deficits at figurative language, including idioms, reflect overall language competence, rather than a specific deficit at this aspect of language [26,54,79,80]. In our study, the children with autism had strong language ability in 4 of 5 cases, but nevertheless appeared to perform differently than their typical and language-impaired peers at online idiom comprehension. It may be that the specific aspect of idiom comprehension that is responsible for this different pattern is not reflected in the standard offline language tests, which measure aspects of grammar (e.g., CELF, TROG) or single word comprehension (e.g., PPVT). This may also reflect a broader difference in methodology: children with autism may appear to have typical language with offline measures, even if online measures reveal differences suggestive of atypical automatic language processing, which nonetheless could still enable high levels of successful comprehension in at least some children.

Despite the clear patterns of results, our study does have some limitations. First, we had only a single test point, and so we cannot draw conclusions about the time course of the availability of the literal *vs.* non-literal meanings. Our results only speak to what is happening at the offset of the final word of the idiomatic expression. Thus we can’t say if, for example, activation of the idiomatic meaning is delayed rather than absent for the children with autism. Second, we included only a single type of idiom, and so our results may not generalize to other idioms that may differ in (e.g.) transparency or familiarity [26]. Third, our determination of idiom familiarity was based on data from college students, not children in the same age range as we tested online. However, there is no reason to believe that younger children would be familiar with idioms that are less familiar to college students, or that young children would perform better with idioms that adults rated as unfamiliar. Finally, we remind readers that this is a within-subjects design, where each participant provides data for all conditions. Even still, while we had a sufficiently large sample for the typical children, we acknowledge the smaller sample sizes for the two disordered groups. Therefore, while we believe the results speak to online processing in these groups, further investigation with larger samples would help to confirm our findings and allow for a more detailed investigation of specific age-related changes in performance for these groups. Despite these limitations however, the results provide information from a novel perspective for all three groups of children.

In conclusion, our results indicate that typically-developing children understand highly familiar idioms and are able to access the meaning of such idioms on-line similarly to adults. Activation of an idiom’s meaning relies on the ability to recognize a configuration of words in an intact semantic memory system. Semantic memory in children with specific language impairment (SLI) may therefore be sufficiently intact to allow a typical priming pattern to emerge – thus not only do children with SLI know the meanings of these idioms, they are able to access them in real time during sentence comprehension. In contrast, children with autism appear to have a particular deficit of semantic memory that does not allow for real time activation of an idiom’s semantic representation.

## Figures and Tables

**Table 1:** Participant information and demographic scores (means and standard deviations).

	Typically developing	SLI	Autism
N	14	7	5
Age (years)	10.6 (2.7)	12.2 (2.4)	11.5 (4.4)
Age Range (years)	6 – 16	9 – 16	8 – 16
Sex	5 M / 9 F	5 M / 2 F	4 M / 1 F
TONI-3	107.7 (16.1)	107.1 (12.4)	117.2 (14.2)
CELF-IV (core language index)	112.3 (11.8)	75.9 (13.0)	109.2 (31.1)
TROG	104.7 (11.5)	82.0 (20.2)	105.6 (22.7)
PPVT-IV	110.9 (12.1)	90.6 (9.6)	123.6 (29.4)
SIDI (CCC-2)	---	6.7 (9.6)	−13.3 (7.6)

*Note*: SLI = Specific Language Impairment; M = Male; F = Female; TON I-3 = Test of Nonverbal Intelligence, 3^rd^ edition; CELF-IV = Clinical Evaluation of Language Fundamentals, 4^th^ edition; TROG =Test for Reception of Grammar; PPVT-IV = Peabody Picture Vocabulary Test, 4^th^ edition. SIDI = Social Interaction Difference Index score from the Children’s Communication Checklist; 2^nd^ edition. Standard scores are presented for standardized tests.

**Table 2:** Pretest results showing the mean (and standard deviation) relatedness of the visual targets to the idiomatic and literal meanings of the idioms.

Idiom (n=40; e.g.: **dished the dirt**)	Literal meaning	Idiomatic meaning
Literal Related (e.g,. **soil**)	**4.28 (.72)**	1.81 (.67)
Literal Control (e.g.. **belt**)	1.31(43)	1.55 (.49)

Idiom Related (e.g.; **gossip**)	1.86 (.48)	**4.45 (.61)**
Idiom Control (e.g.. **duty**)	1.36 (.49)	1.32 (.45)

**Table 3:** Response time (RT) means (and standard error) and priming effects (control-related response times) for each condition for typically developing children, children with *SLI*, and children with autism.

	Idiomatic Meaning		Literal Meaning	
	Control	Related	Control	Related
Typically developing (n=14)				
Mean RT:	1029 ms (17)	973 ms (14)	996 ms (15)	1021 ms (16)
Priming:	51 ms *		−25 ms	

SLI (n=7)				

Mean RT:	896 ms (22)	863 (20)	877 ms (20)	873 ms (13)
Priming*:	33 ms *		7 ms	

Autism (n=5)				

Mean RT:	935 ms (24)	925 ms (20)	967 ms (25)	923 ms (22)
Priming:	10 ms		44 ms*	

* *p*<0.05
